# Anti-Inflammatory Activity of a Polymeric Proanthocyanidin from *Serjania schiedeana*

**DOI:** 10.3390/molecules22060863

**Published:** 2017-05-26

**Authors:** David Osvaldo Salinas-Sánchez, Enrique Jiménez-Ferrer, Veronica Sánchez-Sánchez, Alejandro Zamilpa, Manasés González-Cortazar, Jaime Tortoriello, Maribel Herrera-Ruiz

**Affiliations:** 1 Biomedical Research Center of the South (IMSS), Argentina 1, Col. Centro, Xochitepec 62790, Morelos, Mexico; davidos@uaem.mx (D.O.S.-S.); enriqueferrer_mx@yahoo.com (E.J.-F.); vEra1o@outlook.es (V.S.-S.); azamilpa_2000@yahoo.com.mx (A.Z.); gmanases@hotmail.com (M.G.-C.); jtortora2@yahoo.es (J.T.); 2 Biodiversity and Conservation Research Center (UAEM), Av. Universidad 1001, Col. Chamilpa, Cuernavaca 62209, Morelos, Mexico; 3 Faculty of Biological Sciences (FBC), UAEM, Av. Universidad 1001, Col. Chamilpa, Cuernavaca 62209, Morelos, Mexico

**Keywords:** *Serjania schiedeana*, proanthocyanidins, epicatechin, articulate inflammation, cytokines

## Abstract

The ethyl acetate extract (SsAcOEt) from *Serjania schiedeana*, select fractions (F-6, F-12, F-13, F-14), and one isolated compound, were evaluated in 12-*O*-tetradecanoylphorbol 13-acetate (TPA) ear edema and kaolin/carrageenan (KC)-induced monoarthritis assays. SsEtOAc induced edema inhibition of 90% (2.0 mg/ear), fractions showed activity within a range of 67–89%. Due to the fact F-14 showed the highest effect, it was separated, yielding a proanthocyanidin-type called epicatechin–(4β → 8)–epicatechin–(4β → 8, 2β → O → 7) epicatechin (ETP). This compound (2.0 mg/ear) provoked 72% of edema inhibition (ED50 = 0.25 mg/ear, Emax = 52.9%). After 9 days of treatment, joint inflammation was decreasing, and on the last day, SsEtOAc (400 mg/kg), F-14 and ETP (10 mg/kg), SsEtOAc (200 mg/kg), methotrexate (MTX) 1.0 mg/kg and meloxicam (MEL) 1.5 mg/kg, produced an inhibition articulate edema of 94, 62, 36, 21, 80, and 54%, respectively. In the joint, pro-inflammatory molecules were elevated in animals without treatment (vehicle group, VEH). Treatments from *S. schiedeana* induced a decrease in the concentration of interleukin (IL)-1β, IL-17, and IL-6, and SsEtOAc at a higher dose diminished tumor necrosis factor (TNF-α). IL-10 and IL-4 were fewer in the VEH group in comparison with healthy mice; the animals with treatments from *S. schiedeana* induced an increment in the levels of these cytokines in joint and spleen.

## 1. Introduction

Rheumatoid arthritis (RA) is a chronic systemic inflammatory disease with a worldwide prevalence of 0.3–1% and is associated with significant morbidity and increased mortality. It is more common in women from developed countries with a female:male gender ratio of 3:1 [1]. RA causes pain and deformity, leading to a chronic disabling condition. The World Health Organization (WHO) indicates that within 10 years from the onset of this illness, at least 50% of patients in developed countries are unable to hold a full-time job [2], and this problem affects the middle-age group of 40 ± 10 years.

The main target of RA is synovial membrane, cartilage, and bone, but this disease is capable of giving rise to multi-organic damage, which is related with the chronic and systemic inflammatory scheme. For example, pro-inflammatory cytokines, such as tumor necrosis factor alpha (TNF-α), interleukin (IL)-1β, and IL-6, are released into the systemic circulation and gives rise to alterations in different organs, such as adipose tissue, skeletal muscle, fibrous and connective tissue, tendons, liver, the immune and hematologic systems, and endothelium [3], with the consequent destruction.

Treatment of RA is based on anti-inflammatory drugs, mainly nonsteroidal anti-inflammatory drugs (NSAID), which comprise a heterogeneous group of agents. It is widely accepted that the main mechanism of action of these substances is inhibition of the cyclooxygenase (COX) enzymes (COX-1 and COX-2); these are key in prostaglandin (PG) synthesis. PG regulates the inflammatory mechanisms, among other effects. Other interesting roles for NSAID have been described, for example, inhibition of inducible nitric oxide synthase (iNOS) and nuclear factor kappa Beta (NF-κΒ), a key transcription factor that controls the inducible expression of many genes associated with inflammation, such as TNF-α or IL-1β, and adhesion molecules intercellular adhesion molecule 1 (ICAM-1) and vascular adhesion molecule 1 (VCAM-1). An example of these types of drugs is meloxicam (MEL), which inhibits COX-2. Also, NSAID inhibit phospholipase (PPL) A2 [4,5]. Another type of drug known for being of benefit to RA includes disease-modifying anti-rheumatic drugs (DMARD), such as methotrexate (MTX); these later drugs are prescribed as first choice for RA [6,7], but they also are costly [8]. MTX is a competitive inhibitor of the dihydrofolate reductase enzyme, which catalyzes tetrahydrofolate synthesis; it is necessary for the formation of the thymidylate and purine nucleotides involved in DNA and RNA synthesis. When it is blocked, it gives rise to anti-proliferative effects, thus releasing adenosine and inhibiting polyamines, which participate in the anti-inflammatory effect of this compound. The adverse effects of drugs such as MTX include nausea, stomatitis, ulcers, leukopenia, hepatitis, cirrhosis, and pneumonitis, among other diseases [4]. The severity of these effects impact the patient’s quality of life, and not improving the medical condition of the patient with RA. The study of the medicinal effects of extracts, fractions, or secondary metabolites from plants is an important work objective in the search for novel and better pharmacological treatments of RA.

In Mexico, the species *Serjania schiedeana* (Sapindaceae), commonly known as “bejuco de tres costillas”, is localized in the rainforest denominated “Sierra de Huautla” in the Mexican state of Morelos (Sierra de Huautla Biosphere Preserve [REBIOSH]). This plant is a climbing shrub, the central portion of its stem is woody, and this stem is also surrounded by other external stems (3–5) that are triangular in form. Its leaves are rhomboidal and are densely covered with hairs on both surfaces. The stem of *S. schiedeana* is traditionally used to treat kidney inflammation, burning feet, and back pain, and to heal wounds and bruises [9].

There are, to our knowledge, no data on pharmacological or chemical studies of this species. For the *Serjania* genus, the anti-inflammatory activity has been described by the use of the croton-oil-induced auricular inflammation test in mice. The hydroalcoholic extract and its fractions of *Serjania erecta* with different solvents (dichloromethane, ethyl acetate, and *n*-hexane) in doses ranging from 0.03–3.0 mg/ear) were employed, and results showed an inhibition percentage of between 72% and 83% [10].

Ethnomedical reports of *S. schiedeana* point to its use in inflammatory processes; therefore, the objective of this work was to evaluate the anti-inflammatory activity of the extracts, fractions, and of a major compound from this species, by means of two pharmacologic assays in mice: the 12-*O*-tetradecanoylphorbol-13-acetate (TPA)-induced auricular inflammation test, and arthritis induced by the kaolin/carrageenan (K/C) model. In the latter, cytokine levels were quantified in joints and spleen, while TNF-α, IL-1β. IL-6, IL-17, IL-10, and IL-4 were quantified by the enzyme-linked immunosorbent assay (ELISA) method. 

On the other hand, chemical analysis of the ethyl acetate extract from the branches of the species was performed, and one compound, identified as epicatechin–(4β → 8)–epicatechin–(4β → 8, 2β → O → 7) epicatechin (ETP), was isolated.

## 2. Results

### 2.1. Structural Elucidation of Epicatechin Trimeric Proanthocyanidin (ETP)

**Figure 1:**
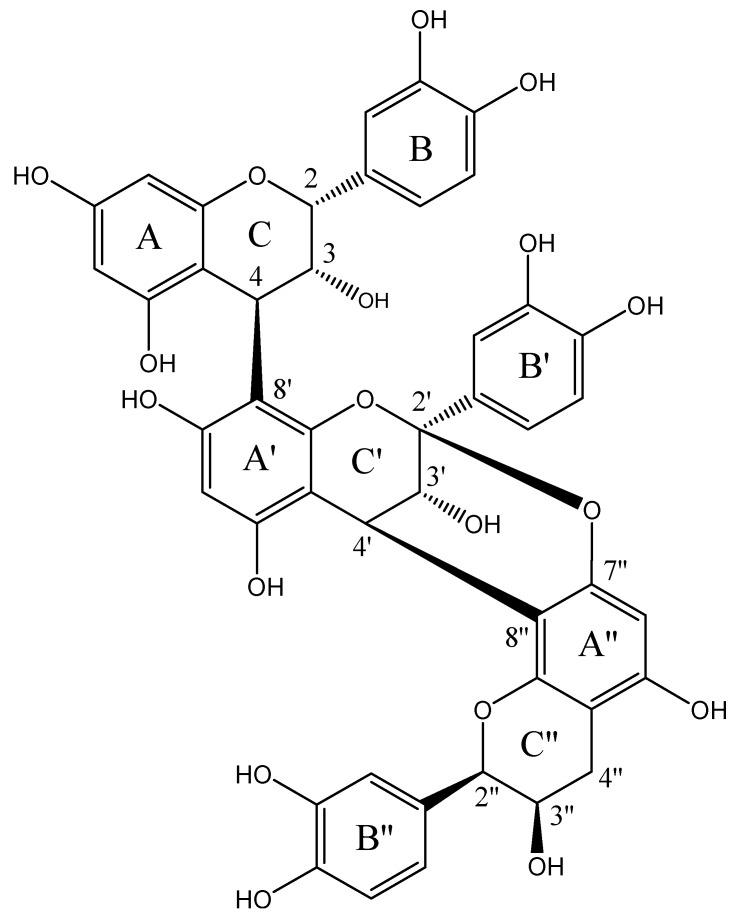
Chemical structure of epicatechin–(4β → 8)–epicatechin–(4β → 8, 2β → O → 7), or epicatechin trimeric proanthocyanidin -ETP-, isolated from the anti-inflammatory fraction F-14 of *Serjania schiedeana*.

The bioactive procyanidin was identified as a polymeric epicatechin derivative: epicatechin–(4β → 8)–epicatechin–(4β → 8, 2β → O → 7) epicatechin (ETP, Figure 1). This chemical identification was based on the comparison of spectrometric (*m*/*z* = 863.11 [M + 1]) as well as one-dimensional (1D) and two-dimensional (2D) nuclear magnetic resonance (NMR) data with those previously reported in Table 1, for this polyphenol previously isolated from cranberry (Table 1) [11,12,13,14,15].

### 2.2. Anti-Inflammatory Activity of Serjania schiedeana in TPA-Induced Ear Edema

Local application of TPA (2.5 µg/ear, vehicle [VEH] group) induced ear edema of 10.3 mg; this effect was inhibited by the administration of 1.0 mg/ear of indomethicin (IND), which gave rise to 88.2% inhibition of inflammation. Both groups were statistically different (*p* < 0.05). The ethyl acetate extract (SsEtOAc, 2.0 mg/ear), F-14, and ETP (1.0 mg/ear) were able to inhibit auricular edema significantly, in comparison with the vehicle group (VEH, *p* < 0.05) (Table 2).

ETP was evaluated at different doses, but only 1.0 and 2.0 mg/ear were able to induce an significant anti-inflammatory effect (47% and 72.2% of inhibition, respectively), with a median effective dose (ED_50_) = 0.25 mg/ear and a maximal effect (Emax) = 52.9%.

### 2.3. Anti-Arthritic Activity

**Figure 2:**
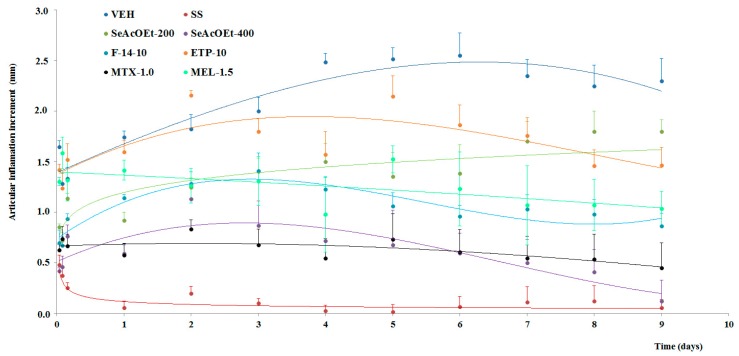
Effect of different treatments of *Serjania schiedeana* on temporary course of joint inflammation of mice exposed to mono-arthritis with kaolin/carrageenan (K/C). SsEtOAc = ethyl acetate extract of *S. schiedeana*; F-14 = a fraction from extract; ETP = epicatechin trimeric proanthocyanidin; MTX = methotrexate; MEL= meloxicam. Data represent the mean of each group (*n* = 12) ± SD.

Evaluation of the evolution of the articulate inflammation was measured as an increment of the edema along 9 days. The group of non-treated animals underwent prolongation of articulate edema, which developed quickly, within hours, and with a duration during all of the days of the assay (●); this effect was statistically different from that of the baseline group (*p* < 0.05), which only received an intra-articulate (i.a.) injection of sterile saline (SS, ●). This group of animals does not exhibit inflammation throughout the assay; a slight increment was only observed during the first hours, but at 24 h after administration of SS, this parameter returned to the baseline value (Figure 2).

Groups with SsEtOAc (400 mg/kg, ●) and fraction F-14 (10 mg/kg, ●) exhibited significant diminution of inflammation from day 1 of the administration of such treatment and up to the final day of the experiment (*p* < 0.05) (Figure 2).

SsEtOAc (200 mg/kg, ●) and ETP (10 mg/kg, ●) induced only slight activity in articulate inflammation by carrageenan, but not until day 5, and this was not significant. The control drugs employed also gave rise to a decrement of edema in mice, MTX (1.0 mg/kg, ●) and MEL (1.5 mg/kg, ●), both groups statistically different from the VEH group (*p* < 0.05) (Figure 2).

The inhibition percentage of the joint edema induced by K/C was calculated on the last experimentation day. At this time, inhibition of local inflammation was 94.6, 62.4, and 80.3% for SeAcOEt 400, F-14, and MTX-1.0, respectively, while the remaining treatments demonstrated low inhibition, with SeAcOEt at 200 mg/kg, ETP at 10 mg/kg, and MEL at 1.5 mg/kg with 21.7, 36.20, and 54.9%, respectively.

**Figure 3:**
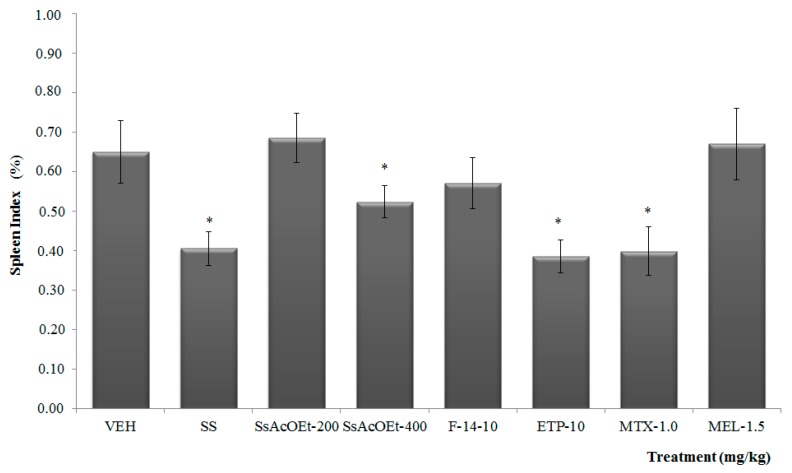
Effect of different treatments of *Serjania schiedeana* on spleen index of mice exposed to mono-arthritis with kaolin/carrageenan (K/C). SsEtOAc = ethyl acetate extract of *S. schiedeana*; F-14 = a fraction from the extract; ETP = epicatechin trimeric proanthocyanidin; MTX = methotrexate; MEL = meloxicam; VEH=negative control. Analysis of variance (ANOVA), post-test Bonferroni (*n* = 12; mean ± SD). * *p* < 0.05 compared with VEH.

The effect of K/C administration was observed on the spleen, which was measured through its weight and calculated the percentage respect to total animal weight. The group with only i.a. K/C showed a significant increase in the spleen index (%) in comparison to the SS group. And animals that received SsEtOAc at 400 mg/kg, ETP, or MTX, presented a decrease in spleen percentage (Figure 3).

### 2.4. Effect of S. schiedeana on Local Cytokines

**Figure 4:**
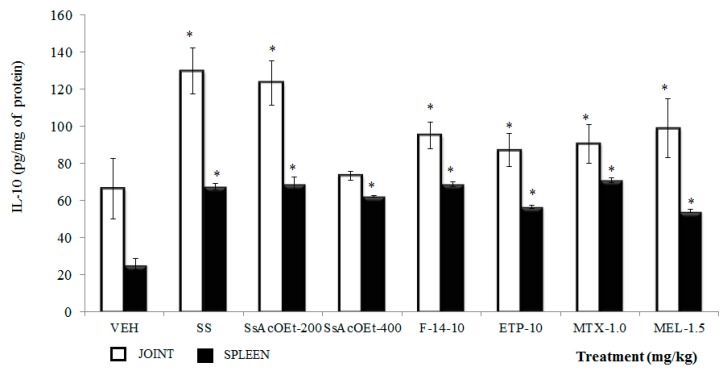
Effect of different treatments of *Serjania schiedeana* on IL-10 concentration in joint and spleen of mice exposed to monoarthritis with kaolin/carrageenan (K/C). SsEtOAc = ethyl Acetate extract of *S. schiedeana*; F-14 = a fraction from extract; ETP = epicatechin trimeric proanthocyanidin; MTX = methotrexate; MEL= meloxicam; VEH=negative control. Analysis of Student *t* test (*n* = 4; mean ± SD). * *p* < 0.05 compared with VEH.

**IL-10**. In Figure 4, the concentration of IL-10 in joint tissue was significantly lower in animals administered only kaolin/carrageenan (K/C), compared to the group with only SS (*p <* 0.05; Figure 4). All treatments (with the exception of SsEtOAc-400), induced a significant increase in IL-10 concentration, with respect to the VEH group (*p* < 0.05). The IL-10 concentration in the spleen of all groups indicated a significantly increment, with respect to the VEH group (*p* < 0.05).

**Figure 5:**
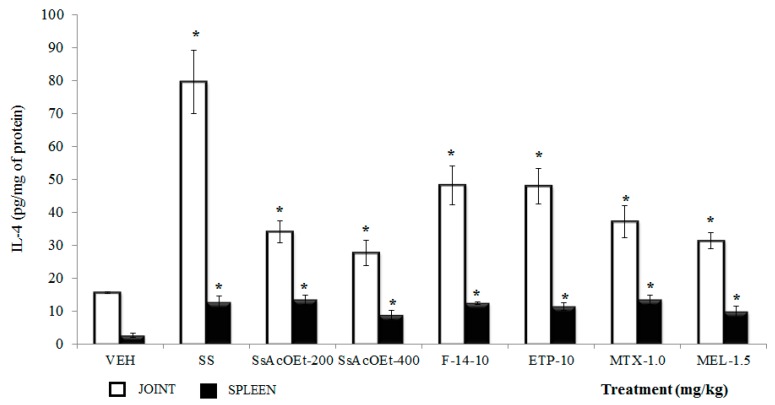
Effect of different treatments of *Serjania schiedeana* on IL-4 concentration in joint and spleen of mice exposed to monoarthritis with kaolin/carrageenan (K/C). SsEtOAc = ethyl Acetate extract of *S. schiedeana*; F-14 = a fraction from the extract; ETP = epicatechin trimeric proanthocyanidin; MTX = methotrexate; MEL = meloxicam; VEH = negative control. Analysis of Student *t* test (*n* = 4; mean ± SD). * *p* < 0.05 compared with VEH.

**IL-4**. All treatments from *S. schiedeana* and drugs utilized as controls caused a significant increase in IL-4 in both joint and spleen in comparison with the VEH group (*p <* 0.05; Figure 5). In joint, the data were not similar to those of the group with SS (*p <* 0.05).

**Figure 6:**
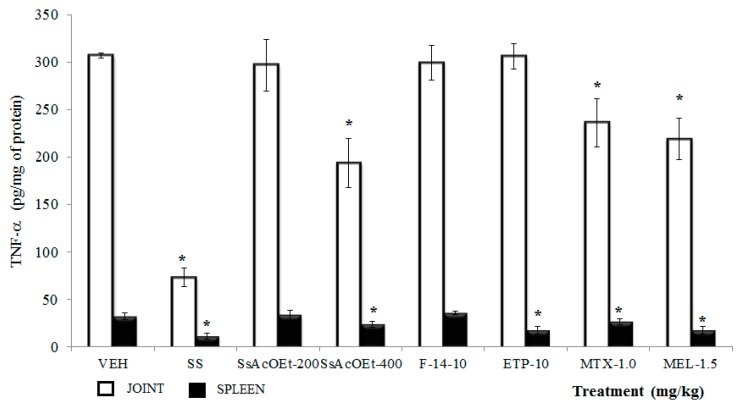
Effect of different treatments of *Serjania schiedeana* on TNF-α concentration in joint and spleen of mice exposed to monoarthritis with kaolin/carrageenan (K/C). SeAcOEt = ethyl Acetate extract of *S. schiedeana*; F-14 = a fraction from the extract; ETP = epicatechin trimeric proanthocyanidin; MTX = methotrexate; MEL = meloxicam; VEH = negative control. Analysis of Student *t* test (*n* = 4; mean ± SD). * *p* < 0.05 compared with VEH.

**TNF-α**. Administration of SsEtOAc at 400 mg/kg, MTX at 1.0 mg/kg, and MEL at 1.5 mg/kg were significantly able to diminish the TNF-α concentration in joint of female mice with i.a. carrageenan (*p <* 0.05). SsEtOAc at 200 mg/kg, F-14, and ETP at 10 mg/kg were not able to counteract the effect of carrageenan in the TNF-α level in joint (Figure 6). For spleen, only treatments of SsEtOAc at 400 mg/kg and ETP at 10 mg/kg together with MEL and MTX induced a significant decrease of this cytokine in comparison with VEH (*p <* 0.05; Figure 6); the remaining treatments failed to do so (*p* > 0.05).

**Figure 7:**
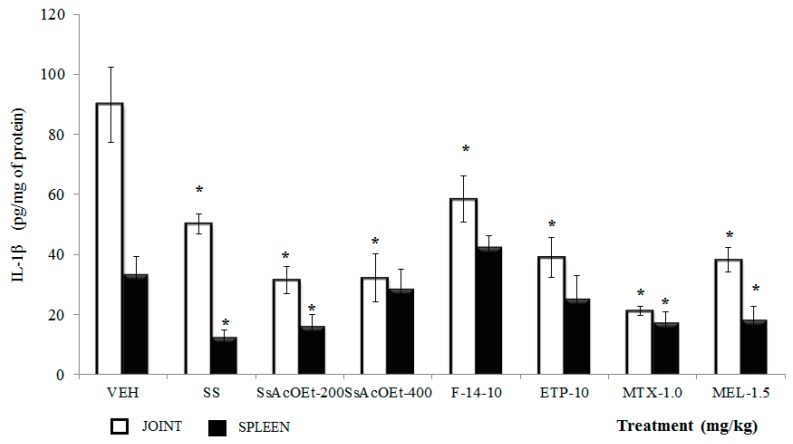
Effect of different treatments of *Serjania schiedeana* on IL-1β concentration in joint and spleen of mice exposed to monoarthritis with kaolin/carrageenan (K/C). SsEtOAc = ethyl acetate extract of *S. schiedeana*; F-14 = a fraction from the extract; ETP = epicatechin trimeric proanthocyanidin; MTX = methotrexate; MEL = meloxicam; VEH=negative control. Analysis of Student *t* test (*n* = 4; mean ± SD). * *p* < 0.05 compared with VEH.

**IL-1β**. In joint from mice of the VEH group, the concentration of IL-1β was superior to those of animals with only SS, and the data were significantly different (*p <* 0.05). Either the treatments administered of *S. schiedeana* (SsEtOAc, F-14, and ETP) or drugs (MEL or MTX) diminished the articulate concentration of IL-1β with respect to that of the control VEH group (*p <* 0.05; Figure 7), while in spleen, IL-1β in mice with only K/C was also elevated in comparison with the SS group. However, not all treatments of the plant were effective for decreasing this cytokine: only SsEtOAc at 200 mg/kg counteracted this effect in this organ (Figure 7). 

**Figure 8:**
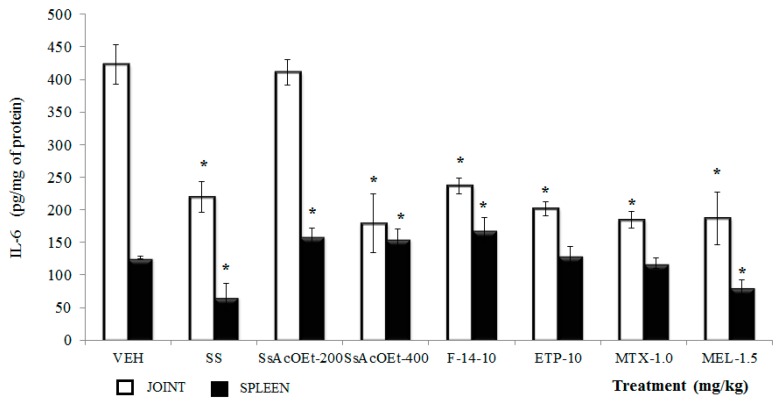
Effect of different treatments of *Serjania schiedeana* on IL-6 concentration in joint and spleen of mice exposed to monoarthritis with kaolin/carrageenan (K/C). SsEtOAc = ethyl acetate extract of *S. schiedeana*; F-14 = a fraction from the extract; ETP = epicatechin trimeric proanthocyanidin; MTX = methotrexate; MEL= meloxicam; VEH = negative control. Analysis of Student *t* test (*n* = 4; mean ± SD). * *p* < 0.05 compared with VEH.

**IL-6**. This cytokine was elevated in joint and spleen of animals of the VEH group with respect to those that only received sterile SS (*p <* 0.05). Administration of SsEtOAc at 200 mg/kg did not modify this result in joint; however, application of SsEtOAc at 400 mg/kg, F-14, and ETP (at 10 mg/kg), with MTX and MEL, induced a decrease in the concentration of IL-6 with respect to the VEH group (*p <* 0.05; Figure 8). However, this behavior was not observed in spleen; instead, for example, for SsEtOAc (two doses) and F-14, there was an increase of this parameter (*p* < 0.05), and only MEL gave rise to a decrease of IL-6 in this organ (*p* < 0.05; Figure 8).

**Figure 9:**
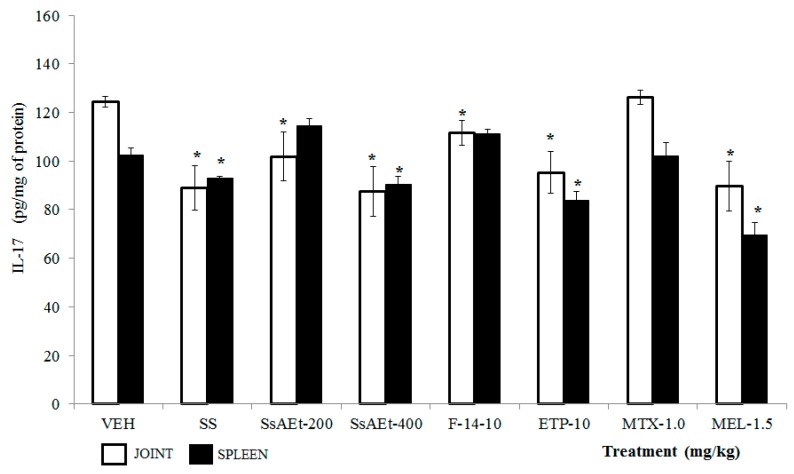
Effect of different treatments of *Serjania schiedeana* on IL-17 concentration in joint and spleen of mice exposed to monoarthritis with kaolin/carrageenan (K/C). SsEtOAc = ethyl acetate extract of *S. schiedeana*; F-14 = a fraction from the extract; ETP = epicatechin trimeric proanthocyanidin; MTX = methotrexate; MEL = meloxicam; VEH = negative control. Analysis of Student *t* test (*n* = 4; mean ± SD). * *p* < 0.05 compared with VEH.

**IL-17**. This cytokine was elevated in joint and spleen of mice that only received VEH, and data were different from those of the SS group (*p <* 0.05). The complete extract of SsEtOAc (200 and 400 mg/kg), F-14, and ETP demonstrated a significant decrease in the concentration of IL-17. For spleen, only the higher dose of SsEtOAc and ETP demonstrated a significant decrease. In the case of the drugs, only MEL at 1.5 mg/kg diminished this cytokine in both organs (*p <* 0.05; Figure 9).

## 3. Discussion

In traditional Mexican medicine, the use of species of the genus *Serjania* is relevant. They are utilized for the treatment of diseases whose general physiopathological background is inflammation [9]. Despite their extended use the Mexican Republic are few reports in the literature on the pharmacological evaluation of the species of this genus, which are widely distributed in Mexico. In particular, concerning the species *Serjania schiedeana*, there are, to our knowledge, no scientific references of its pharmacology and/or chemistry. In the present study, we conducted a pharmacological study on the anti-inflammatory activity of the ethyl acetate extract of the species and of its fractions, employing two assays: first, that of (TPA)-induced auricular edema, and the second, experimental mono-arthritis induced with kaolin/carrageenan (K/C).

The results presented herein indicate that *S. schiedeana* possesses anti-inflammatory activity in the test of auricular edema with TPA. With thin-layer chromatography (TLC) using the Komarovsky and NP-PEG reagents revealed that fractions F-6, F-12, and F-13 contain flavonoids and terpenes and cathechins. However, in the fraction with greatest biological activity, F-14, epicatechin-type compounds predominate, as in the case of ETP. TPA extracted from croton oil, when applied locally, produces edema caused by means of leukocyte infiltration, mainly neutrophils. In addition, it induces the expression of the nitric oxide synthase enzyme through the activation of protein kinase C in different tissues and pro-inflammatory cells. TPA is able to prompt the release of eicosanoids, and stimulate the enzyme phospholipase A2, which causes the release of arachidonic acid and prostaglandins, as PGE2 [9,16]. There are no reports about pharmacologic activity of *S. shiedeana*, but it was reported that extracts and fractions from *S. erecta* are capable to reduce of croton-oil ear edema [10]. Other species from the same family, *Dodonaea polyandra* possess secondary metabolites with anti-inflammatory activity in TPA edema test, like the terpenes 15,16-epoxy-8α-(benzoloxy)methyl-2-oxo-cleroda-3,13(16),14-trien-18-oic acid, 15,16-epoxy-8α-(benzoloxy)methyl-2α-hydroxycleroda-3, 13(16),14-trien-18-oic acid, and 15,16-epoxy-2α-benzoloxycleroda-3,13(16),14-trien-18-oic acid [17,18]. From *D. viscosa*, the clerodane-type terpene hautriwaic acid (HA) was isolated, which exhibited anti-inflammatory activity on the same assay in mice, in an acute as well as in a chronic phase [19]. And also, the anti-arthritic, in the K/C-induced model of mono-arthritis, of this terpene (to 5, 10, and 20 mg/kg) was reported [20]. 

Due to that F-14 from *S. schediana* induced greater activity in the TPA assay, it was chemically separated and achieved the isolation of a major compound whose spectroscopic characteristics indicate that it is an oligomeric proanthocyanidin of three units (ETP). And the evaluation of different doses de ETP employing the TPA model, showed a dose-dependent anti-inflammatory effect, with significant activity at the doses of 1.0 and 2.0 mg/ear. From the group of catechins, it has been demonstrated that the mixture of theaflavins obtained from black tea, such as theaflavin-3-gallate, theoflavine-3′-gallate, and theoflavin-3,3′-digallate, significantly inhibit TPA-induced ear edema, not only in an acute manner, but also after an application of the irritant for 4 days, through a mechanism in which is involved in the inhibition of the metabolism of arachidonic acid (AA) by blocking the cyclooxygenase and lipoxygenase enzymes [21,22,23]. 

The extract, fraction F-14 and ETP were evaluated in the experimental mono-arthritis assay induced with K/C and was observed a decrement of joint inflammation along to 9 days of experiment. Under these conditions, the administration of any of the treatments deriving from *S. schiedeana* caused a significant diminution of cytokines IL-1β and IL-17 (both comprise key factors of inflammation during RA), and also of IL-6 on joint tissue. With respect to TNF-α, only SsEtOAc at 400 mg/kg could reduce the levels of this cytokine in joints; the remaining treatments did not produce changed in this protein. For the anti-inflammatory cytokines, the groups that received some treatment of the plant presented a significant increase in the joint concentration of IL-10, as well as of IL-4, with the exception of the group that received a high dose of SsEtOAc, in which we did not observe changes with regard to the VEH group.

Cytokines participate in the establishment and maintenance of an inflammatory state in RA. The i.a. administration of K/C leads to the production of the pro-inflammatory cytokines that are characteristic of the arthritic process, including TNF-α, IL-1β, IL-6, and IL-17. These results confirm the active participation of lymphocytes and macrophages, which constitute the main producers. Among the inflammatory process’s measures of contention, we find IL-10 and IL-4; both cytokines are found at low concentrations in the joints of patients with RA. IL-10, as well as IL-4, possesses the capacity to regulate the production of IL-1β and TNF-α due to their effect on monocytes, and especially on macrophages. This also inhibits the production of MMP-1, inducing the expression of its inhibitor. Both cytokines are capable of preventing the destruction of joint tissue [24]. In the present description, it can be observed that mice that received K/C administered i.a. and, as treatment, VEH alone, exhibited a significant increase in the concentrations of all of the cytokines considered as pro-inflammatory (TNF-α, IL-1β, IL-6, and IL-17), in addition to low levels of IL-10 and IL-4 in joints and in spleen, this in comparison with healthy animals. 

In synovial tissue, TNF-α promotes the secretion of IL-1β, IL-6, and IL-8; in vitro studies suggest that TNF-α, similarly to IL-17, is important due to its place in the hierarchy of the cascade that regulates the production of pro-inflammatory cytokines in rheumatoid synovial tissue [25,26,27]. Among its multiple effects on articulation, may include the increase in the expression of adhesion molecules, prostaglandin PGE2, and the stimulation of the chondrocytes to produce matrix metalloproteinases (MMP), enzymes that participates with in the destruction of joint cartilage. TNF-α is produced mainly by macrophages in the synovial membrane and in the cartilage-pannus junction [24]. In the K/C-induced model of arthritis, the levels of this cytokine in the group with damage arose to four-fold more compared with the group with normal recovery from inflammation (SS). As mentioned previously, the abundance of this cytokine suggests an inflammatory state, both systemic (spleen) and local (joint), that lasts during days. Administration of extract from *S. schediana* improves this condition, probably due to the presence of terpenes, flavonoids, and catechins that acting in conjunction on multiple points of the chronic inflammatory process, favoring the diminution of TNF-α.

IL-1β shares some biological functions with TNF-α, in that it is a potent inducer of damage in cartilage and bone on in turn inducing the degradation of proteoglycans, inhibiting their synthesis and activating bone resorption by osteoclasts; additionally, the production of MMP and collagenases is mediated by this cytokine. IL-1β and TNF-α, are found at elevated concentrations in the synovial tissue of persons with RA [25]. In the model employed, the concentrations of IL-1β are elevated, an effect that is counteracted by SsEtOAc, F-14, and ETP treatments that significantly diminish the levels of the pro-inflammatory cytokine in joint and spleen.

Another cytokine that has been related with the physiopathology of RA is IL-6, which is produced principally by fibrobast-type synoviocytes and macrophages. IL-6 appears to exert different actions on different cellular types, with effects that are pro-inflammatory as well as anti-inflammatory. In RA, IL-6 mainly favors the inflammation through its influence on T and B lymphocytes, the expression of chemokines in endothelial cells, and the activation of osteoclasts [28]. The concentration of this cytokine is elevated in synovial fluid and in the tissue of persons with RA [29,30]. In the present work, animals that received VEH as treatment, IL-6 concentrations were greater than those observed in SS. The SsEtOAc fraction of *S. schiedeana* at 400 mg/kg, F-14, and ETP diminished the concentration of this cytokine in the joints.

IL-17 is an important factor of differentiation for Th17 cells; it is thought that its effect on RA is crucial in the disease’s early phases, to a greater degree than in the cellular effector phases. IL-17 has been found in the synovial tissue of individuals with arthritis; it is a pro-inflammatory cytokine and possesses a primary role in the physiology of RA [28,31]. The SsEtOAc, F-14, and ETP treatment of *S. schiedeana* were capable of diminishing the levels of IL-17 in the synovium. This demonstrated that the plant possesses the capacity to improve the condition of damage of the animals by modulating the local concentration of different inflammatory mediators, like IL-17, IL-1β, IL-6 and TNF-α.

On the other hand, it has been demonstrated that in KO mice with experimental arthritis-induced with collagen (type II) presented high expression of IL-17 and the synovial concentrations of this interleukin it was increased up to 900 pg/mg. But in the presence of IL-10 the expression of the messenger RNA (mRNA) of IL-17, and the protein diminished significantly [32]. Then, it is probably that the effect of extracts of *S. schediana*, could be due to indirect activity on IL-17, caused by the increase of IL-10. IL-4 and IL-10 molecules are defined as the products of Th2-cell activation in mice, and their anti-arthritic activity has been demonstrated. These cytokines have the capacity of suppressing and counterbalancing the pro-inflammatory response mediated by Th-1 cells in different disorders. IL-4 inhibits Th1 activity and inflammation in RA and restores the Th1/Th2 disequilibrium in this disease. IL-10 exerts a similar effect, although this is not considered a specific product of Th2 in humans, in that it is also produced by B cells, monocytes/macrophages, and Th-1 cells [33].

Treatments derived from *S. schiedeana* induce clear diminution of the factor of damage of experimental RA, which includes local and systemic inflammation, whose response is associated with changes in the joint and spleen concentration of some cytokines. It is possible that the plant exerts a modulatory effect, and that this is concerned with the chemical complexity of the treatment administered. For example, the complete extract at a low dose does not modify TNF-α and IL-6 levels in joints (the Th1 response). However, it does increase IL-10 and IL-4 (Th2 response), while the 400-mg/kg dose of this same treatment diminishes all of the pro-inflammatory cytokines and raises IL-4, without modifying IL-10. The F-14 fraction and the ETP compound exhibit similar behavior at the highest dose, but these treatments give rise to the two anti-inflammatory cytokines, in joints as well as in spleen. 

In terms of the response to inflammation, it has been observed that the spleen responds, for example, to repeated infections, hyper-function in the generation of lymphoid cells and of antibodies, in order to counteract the damage, which leads the organ to an increase in size. In a study in which the assay was employed of plantar edema induced with carrageenan, the authors demonstrated that the spleen underwent hypertrophia [10]. In another study, it was evidenced that in Tnf−/− mice induced with experimental RA with collagen type II/complete Freud’s adjuvant (CFA), the mice exhibited a significant increase in spleen size in comparison with that of wild-type [34]. This organ combines the innate immune and adaptive responses in a unique organized pathway. Mediators of the inflammation play an outstanding role in this organ: TNF-α is involved in the functioning of the organ itself, and an increase in this cytokine results in splenic dysfunction and tissue damage; additionally, the suppression of TNF-α in spleen could diminish the immune reaction. Treatments derived from *S schiedeana*, SsEtOAc 400 and ETP produced a significant diminution of this protein, indicating suppression at the systemic level. This coincides with the fact that the splenic index of animals that received these treatments was lower than VEH group. Diminution of TNF-α would be a consequence of the fact that IL-1β and IL-6 in this lymphoid organ would also be observed as diminished, because TNF-α is an activator of the production of both of these.

Was observed a diminution of IL-1β in spleen with the administration of SsEtOAc 200 (a dose that does not produce diminution of the splenic index nor of the concentration of the TNF-α); the remaining treatments did not modify this cytokine with respect to the VEH group in that organ. Some data show that while TNF-α is important in the generation of joint damage during RA, it is not essential and that, in the production cascade of the inflammatory mediator, there is no strict correlation between the presence of TNF-α and the secretion of other immunological products [34].

The anti-inflammatory effect caused by *S. schiedeana* is based on the chemical composition of the extract and its fractions. In this chemical study, a trimer of epicatechin (epicatechin–(4β → 8)–epicatechin–(4β → 8, 2β → O → 7) epicatechin) was isolated. This group of compounds have shown anti-arthritic capacity. For example, epigallocatechin gallate (EGCG at 50 mg/kg/day/10 days) diminished synovial fibroblasts, the activity of the transforming growth factor β-activated kinase 1 (TAK1), a key protein in the signaling of IL-1β and TNF-α, in animals with experimental RA induced with CFA [35]. 

## 4. Materials and Methods

### 4.1. General Information

12-*O*-Tetradecanoylphorbol 13-acetate (TPA), indomethacin (IND, ≥99%, TLC), carrageenan (C, a plant mucopolysaccharide), methotrexate (MTX, ≥98%, HPLC), meloxicam sodium salt hydrate (MEL, ≥98%, HPLC), and acetonitrile were purchased from Sigma Chemical Co. (St. Louis, MO, USA). Aluminum silicate (kaolin) was obtained from Merck KGaA (Darmstadt, Germany). The origin of the IL-1β, TNF-α, IL-6, IL-17, IL-4, and IL-10 cytokine kits was BD Biosciences, Inc. (San Diego, CA, USA). The solvents methanol, acetone, dichloromethane (DCM), and ethyl acetate (EtOAc) were purchased from Mallinckrodt Baker, Inc. (Phillipsburg, NY, USA). The organic whole extracts, as well as the subfractions, were separated and analyzed by the application of traditional chromatographic methods such as column chromatography and thin-layer chromatography (TLC), using the Komarovsky and NP-PEG reagents. The silica gel (70–230 mesh), the reverse-phase silica gel C18 (40–63 μm, Merck), and the chromatographic plates were acquired from Merck KGaA. Extracts and fractions of the plant species were separated by column chromatography and monitored by TLC. Normal phase silica gel (70–230 mesh) and reverse phase (RP-18, 40–63 μm, Merck), chromatographic plates (CP), and 1% serum ammonium sulphate in H_2_SO_4_ 2N were obtained from Merck KGaA. The developers used were 2-aminoethyldiphenyl borinate (detection of flavonoids) and 4-hydroxy-benzaldehyde (detection of terpenes). The isolate of ETP was subjected to nuclear magnetic resonance (NMR) analysis on a spectrophotometer, which was recorded at 298 K in a Inova 400 MHz (9.4 T) spectrometer (Varian, Palo Alto, CA, USA) operated at 400.13309 MHz for ^1^H-NMR and at 100.61 MHz for ^13^C-NMR. Tetramethylsilane (TMS) as internal standard. Spectrometric analysis was performed on a Waters Xevo TQD mass spectrometer with ESI ion source (Waters Corp., Milford, MA, USA). Several spectra are displayed in the supplementary material.

Chromatographic analysis by HPLC was performed on a Waters 2695 Separation module system equipped with a Waters 996 photodiode array detector and Empower Pro software (Waters Corporation). Chemical separation was achieved using a Supelcosil LC-F column (4.6 mm × 250 mm i.d., 5-µm particle size, Sigma-Aldrich, Bellefonte, PA, USA). The mobile phase consisted of 0.5% trifluoroacetic acid aqueous solution (solvent A) and acetonitrile (solvent B). The gradient system was as follows: 0–1 min, 0% B; 2–4 min, 10% B, 5–7 min, 20% B; 8–14 min, 30% B; 15–18 min, 40% B; 19–22 min, 80% B; 23–26 min, 100% B; 27–28 min, 0% B. The flow rate was maintained at 0.9 mL min^−1^, and the sample injection volume was 10 µL.

### 4.2. Plant Material

The stems from *Serjania schiedeana* were collected at REBIOSH and a sample was deposited at the Autonomous University of the State of Morelos Herbarium (HUMO) with registry no. HUMO-23690. Identification was conducted by Botanist Juan Carlos Juárez, B.Sc., of the Cuernavaca, Morelos-based Center for Research in Biodiversity and Conservation (CIByC).

### 4.3. Preparation of Extracts

The dried and milled material (2 kg) was extracted with methanol (5 L × 3 times) for 3 days; the solvent was completely removed by distillation under reduced pressure with a rotary evaporator (Heidolph Laborota 4000, Heidolph Instruments GmbH & Co. KG, Schwabach Germany), yielding 221 g (11.05%). This extract was fractionated by the partitioning process using water and ethyl acetate. Aqueous (SsAq) and ethyl acetate (SsEtOAc) fractions were obtained in a yield of 38 and 7.3%, respectively, with respect to SsMeOH.

### 4.4. Epicatechin Trimeric Proanthocyanidin (ETP) Isolation

The most anti-inflammatory fraction (SsEtOAc, 15 g) was fractionated on a chromatographic column (4 × 50 cm) previously packed with 100 g of silica gel (60–230 mesh, from Merck, Whitehouse Station, NJ, USA). An acetone-methanol gradient system was used as mobile phase, starting with 100% of the less polar solvent and ending with 100% methanol. The entire procedure was monitored by TLC. From this process, four anti-inflammatory fractions, including F-6, F-12, F-13, and F-14, were obtained, among which F-14 (3.75 g, 25%) exhibited highest activity. Chemical separation of this latter mixture (3.0 g) was purified under reversed-phase chromatographic column (10 g silica gel RP-18, 40–63 μm, Merck). Mobile phase consisted of the water/acetonitrile gradient system (samples of 10 mL each). The major compound (ETP) obtained from the chromatographic process (F14-9, 191 mg) was identified by NMR (^1^H, ^13^C, COSY, HSQC, and HMBC).

### 4.5. Pharmacological Activity

#### 4.5.1. Animals

CD-1 female mice were used, each weighing 25–30 g. They were provided by the Production and Experimentation Unit of Laboratory Animals (UPEAL) of the Xochimilco Metropolitan Autonomous University (UAM, Xochimilco) in Mexico. The experiments were carried out according to Official Mexican Norm 062-ZOO-1999 (Technical Specifications for the Production, Care and Use of Laboratory Animals) and the international ethical guidelines for the care and use of laboratory animals. The experimental protocol was authorized by the local Health Research Committee (Mexican Institute of Social Security [IMSS]. The animals were maintained at a temperature of 22 ± 3 °C, at a humidity of 70 ± 5%, and under 12 h–12 h light–dark cycles, with water and food ad libitum. In the arthritis bioassay, a control group was utilized; the animals of this group that received a K/C injection in knee joint and oral pathway (o.p.) administration of the vehicle (VEH) without any drugs (the negative control). There were two positive control groups: one administered with the drug meloxicam (MEL), and the other group, with methotrexate (MTX) and intra-articular (i.a.) injection of K/C. A baseline group of healthy animals was also employed; these animals were injected into the knee with sterile saline solution [SS]. CD-1 female mice were also used for TPA-induced auricular edema, as describe in the next section Section 4.5.2.

#### 4.5.2. Model of Acute Inflammation in Mice with TPA

Mice were grouped as eight individuals and the ear inflammation was induced following the previously described method of Payá [36]. First 2.5 μg of TPA was dissolved in 20 μL of acetone, and then 10 μL was applied on the internal and other 10 μL on the external surfaces of the right ear to cause edema. All treatments were dissolved in acetone and also applied topically on both sides (10 μL) of the right ear immediately after administration of TPA. The left ear received acetone as vehicle (VEH) in the same scheme as treatments. Treatments included: SsEtOAc, evaluated at 2 and 4 mg/ear, and F-6, F-12, F-13, and F-14, evaluated at 1.0 mg/ear, and ETP at doses of 0.125, 0.25, 0.5, 1.0, and 2.0 mg/ear. The reference anti-inflammatory drug (IND) was administered at 1.0 mg/ear. 

Six hours after administration of the inflammatory agent, the animals were sacrificed by cervical dislocation. Circular sections 6 mm in diameter were taken, which were weighed. The results were used to determine the TPA-induced edema and to calculate the inhibition percentage by using expression below:

Inhibition % = (∆w control − ∆w treatment/∆w control)·(100)

where ∆w = wt − wnt; wt is the weight of the section of the treated ear; wnt is the weight of the section of the non-treated ear.

#### 4.5.3. Arthritis Model Induced by Kaolin/Carrageenan (K/C)

This experimental design lasted 15 days. There were eight groups of 12 mice; each received its treatment by oral pathway (o.p.). With the exception of healthy mice, all groups were administered by i.a. with kaolin/carrageenan (K/C):

Group 1: healthy mice (SS)
Group 2: SsEtOAc at 200 mg/kg (SsEtOAc-200)
Group 3: SsEtOAc at 400 mg/Kg (SsEtOAc-400)
Group 4: F-14 at 10 mg/kg (F-14-10)
Group 5: ETP at 10 mg/kg (ETP-10)
Group 6: Methotrexate at 1.0 mg/kg (MTX-1.0)
Group 7: Meloxicam at 1.5 mg/kg (MEL-1.5)
Group 8: Negative control group (NO treatment, only K/C-VEH-).

First, measurements were taken from the baseline size of right knee (RK) with a Mitutoyo-brand digital micrometer. Then, the animals of Groups 1–5 were anesthetized with pentobarbital sodium administered intraperitoneally (i.p,) at a dose of 55 mg/kg. Later, these mice were slowly injected with a 40 µL of kaolin (K, 4% solution) in the joint cavity of the RK. Consecutively, flexions and extensions were performed during 15 min in the joint administered with the drug. Immediately after 15 min of flexions, the mice were injected in the same right-joint cavity with 40 µL of carrageenan at 2%. Similarly, flexions were carried out for additional 5 min. Administration of the treatments was conducted with o.p. and was initiated 24 h after damage induction. Afterward, joint edema was measured every 24 h, and on day 9, the mice were sacrificed and suspended from a surgical table to dissect the RK and spleen of each animal. 

#### 4.5.4. Spleen Index

The spleen was weight, and “spleen index” was calculated with respect to the total weight of the animal and represented as percentage.

#### 4.5.5. Homogenization of Joint and Spleen Tissues

After the sacrifice, joint and spleen organs were frozen at −70 °C for later homogenization and determination of the pro-inflammatory cytokines (TNF-α, IL-1β, IL-6 and IL-17) and anti-inflammatory IL-10 and IL-4 by the enzyme-linked immunosorbent assay (ELISA) method.

The RK was thawed to a temperature of 4 °C, at which time the samples were manipulated for their analysis. The tissue was placed in a mortar and covered with dry ice. Then, the tissue it was pulverized and completely disintegrated until the dry ice was sublimated. Separately, samples of articulation and samples of spleen from 3 animals were placed in a vial and disintegrated with 2000 µL of phosphate buffered saline (PBS) (pH 7.4) with phenylmethyl sulfonyl fluoride (PMSF) at 0.01% dissolved in isopropyl alcohol. To completely homogenize the tissues, it was employed an Ultra Turrax T-10 Homogenizer (Cole Parmer, Vernon Hills, IL, USA) during 15 s. This was allowed to rest for 30 s, and the procedure was repeated five additional times. Afterward, the samples were placed in a centrifuge at 12,000 rpm during 5 min. We obtained 300-µL aliquots in the centrifuged microtubes. These were immediately stored at −70 °C for their later use in the ELISA method and served to quantify the different cytokines (*n* = 4, that is to say that from 12 animals in each group were obtained four samples).

#### 4.5.6. Quantification of Cytokines IL-1β, IL-6, IL-17, TNF-α, IL-4, and IL-10 by ELISA

Quantification of each of these cytokines was carried out by the ELISA method utilizing a kit (OptEIATM ELISA sets; BD Biosciences, Franklin Lakes, NJ, USA) and following the manufacturer’s instructions. Briefly, to 96-well plates, we added 100 µL/well of the antibody uptake; the plates were incubated for 12 h at 4 °C. Once this time had elapsed, the plate was washed with PBS buffer (0.05% of Tween-20, 300 µL/well × 3 times). We added 100 µL of PBS with fetal bovine serum (FBS) at 10%, pH 7.0, during 1 h at room temperature. The contents were discarded and the plate was washed with PBS buffer (0.05% of Tween-20, 300 µL/well × 3 times). To the corresponding wells, we added 100 µL of the standard, the target (PBS with FBS), and the test samples. The plate was incubated for 2 h at room temperature. The contents were discarded and the plate was washed with PBS buffer (0.05% of Tween-20, 300 µL/well × 5 times). For TNF-α, IL-6, IL-4, and IL-10, we added 100 µL/well of detection antibody, and streptavidin-horseradish peroxidase (HRP) enzyme. These plates were incubated for 1 h, and washed with 300 µL/well × 7 times, with a PBS solution (added with 0.05% of Tween-20).

For IL-1β, we added 100 µL/well of antibody detection; this was incubated for 1 h and washed with 300 µl/well × five times, with a solution of PBS (added with 0.05% of Tween-20) followed by the streptavidine-HRP enzyme (100 µL/well). These plates were incubated for 1 h, and washed with 300 µL/well × 7 times, with a PBS solution (added with 0.05% of Tween-20).

To each well it was added 100 µL of *o*-phenylendiamine (OPD) substrate that had been previously prepared (one tablet of OPD and one of urea dissolved in 20 mL of distilled water). This was incubated for 30 min at room temperature under conditions of total darkness, and we then added a stop solution (2N H_2_SO_4_). Reading of the plate was carried out in a Stat Fax 2100 Spectrophotometer (Awareness Technologies, Bellport, NY, USA) at a 450-nm wavelength at 37 °C.

For IL-17 was added 50 µL/well of RD1–38 and 50 µL/well of a standard into the corresponding wells. The plate was gently shaken for 1 min and was incubated at room temperature for 2 h. After this, the contents were discarded and the plate was washed with PBS buffer 5 times. We added 100 µL of polyclonal antibody peroxidase to each well and incubated this again for 2 h at room temperature, with consequent washing five times. Then, we added 100 µL to substrate solution and incubated this for 30 additional min in total darkness. Finally, we added stop solution (100 µL, 2N H_2_SO_4_) to each well with gently shaking. Reading of the plate was at a 450-nm wavelength at 37 °C.

#### 4.5.7. Statistical Analysis 

Statistical analysis was performed with an SPSS ver. 17.0 software program (SPSS Inc. Released 2008. SPSS Statistics for Windows, Version 17.0. Chicago: SPSS Inc., Chicago, IL, USA) and the results were expressed as mean ± standard deviation (SD). Data from TPA and spleen index assays, were analyzed by using Analysis of variance (ANOVA) followed by the post hoc Bonferroni test (* *p* < 0.05), and Student *t* test was used to analyze the results of cytokines measure. 

## 5. Conclusions

The anti-inflammatory and modulatory effect of the immune response caused by *Serjania schiedeana* is based on the chemical composition of the extract and its fractions. The bioguided chemical study permitted the isolation and structural identification of a trimer of epicatechin: epicatechin–(4β → 8)–epicatechin–(4β → 8, 2β → O → 7) epicatechin (ETP) with antiarthritic properties. To our knowledge, this is the first time that a proanthocyanidin has been reported in the genus *Serjania*. This scientifically corroborates the traditional use given to this species by the population of the Biósfera Sierra de Huautla in the state of Morelos, Mexico.

## Figures and Tables

**Table 1:** ^1^H- and ^13^C-Nuclear Magnetic Resonance (NMR) data (400 and 100 MHz, CD_3_OD) of Epicatechin Trimeric Proanthocyanidin (ETP).

Ring	No.	^13^C	^1^H	Ring	No.	^13^C	^1^H	Ring	No	^13^C	^1^H
C	2	79.01	5.7, s, m	C′	2′	105.08	3.28 (d, 3.47) 4.15 (d, 3.47)	C″	2″	80.44	4.39 (s)
	3	72.72	4.13, d, m		3′	67.33	3.28 (d, 3.47)		3″	67.67	3.87–3.85 (m)
	4	38.92	4.6, br, s		4′	29.01	4.15, (d, 3.47)		4″	30	a. 2.84 (dd, 4.6, 17.3) b. 2.84 (dd, 4.6, 17.3)
A	5	156.90		A′	5′	151.2		A″	5″	156.16	
	6	98.9	6.02 (d, 2.3)		6′	96.21	5.8 (s)		6″	96.61	6.10 (s)
	7	157.95			7′	155.9			7″	155.94	
	8	96.7	5.98 (d, 2.3)		8′	106.87			8″	108.9	
	9	154.29			9′	151.92			9″	151.92	
	10	106.55			10′	100.09			10″	100.19	
B	11	131		B′	11′	119.56		B″	11″	133.3	
	12	116.86	7.32 d (1.9)		12′	115.88	6.10 d (1.5)		12″	115.85	7.03 (d, 1.93)
	13	145.89			13′	145.46			13″	146.74	
	14	146.42			14′	145.89			14″	146.03	
	15	120.01	6.83 (d, 8.0)		15′	116.14	6.83 (d, 8.0)		15″	116.27	6.75 (d, 8.0)
	16	121.49	7.2 (dd, 1.9, 8)		16′	115.61	7.2 (dd, 1.9, 8)		16″	119.56	6.73 (dd, 1.93, 8.04)

**Table 2:** Effect produced by the extract and fractions obtained from *Serjania schiedeana* on auricular edema induced by TPA (2.5 μg/20 μL) in mice.

Treatment	Doses (mg/Ear)	Edema (mg) Mean ± SEM	Edema Inhibition (%)	Pharmacologic Parameters
SsEtOAc	2.0	1.02 ± 0.71 *	90.09	
F-6	1.0	2.94 ± 0.34 *	71.68	
F-12		2.24 ± 0.70 *	78.42	
F-13		3.40 ± 1.0 *	67.24	
F-14		1.04 ± 0.22 *	89.95	
ETP	0.125	7.9 ± 1.01	23.5	ED_50_ = 0.25 mg/ear Emax = 52.9%
	0.25	7.6 ± 0.73	26.46	
	0.5	7.5 ± 0.89	27.74	
	1.0	5.5 ± 1.39 *	47.01	
	2.0	2.8 ± 0.93 *	72.22	
IND	1.0	1.2 ± 0.4 *	88.25	
VEH	----	10.3 ±1.46	----	

Analysis of variance (ANOVA) with post hoc Bonferroni (*n* = 8; mean ± SD). * *p* < 0.05 in comparison with VEH group (TPA).

## Supplementary Materials

Supplementary materials are available online.
