# Supplementary material for: Anti-Inflammatory Activity of a Polymeric Proanthocyanidin from Serjania schiedeana
Source: Molecules. 2017 May 26;22(6):863. doi: 10.3390/molecules22060863 (PMC6152783; doi:10.3390/molecules22060863)
Supplement: Supplementary file 1 [file molecules-22-00863-s001.pdf]

Anti-inflammatory activity of a polymeric proanthocyanidin from *Serjania schiedeana*

David Osvaldo Salinas-Sánchez [1,2], Enrique Jiménez-Ferrer [1], Verónica Sánchez-Sánchez [1,3], Alejandro Zamilpa [1], Manasés González-Cortázar [1], Tortoriello J [1] and Maribel Herrera-Ruiz [1,*]

1 Biomedical Research Center of the South (IMSS), Argentina 1, Col. Centro, Xochitepec 62790, Morelos, Mexico; E-Mails: enriqueferrer_mx@yahoo.com (E.J.-F.); azamilpa_2000@yahoo.com.mx (A.Z.); gmanases@hotmail.com (M.G.-C.)

2 Biodiversity and Conservation Research Center. (UAEM), Av. Universidad 1001, Col. Chamilpa, Cuernavaca 62209, Morelos, Mexico; E-Mail: davidos@uaem.mx (D.O.S.-S.)

3 Faculty of Biological Sciences (FBC), UAEM, Av. Universidad 1001, Col. Chamilpa, 62209 Cuernavaca, Morelos, Mexico; E-mail: vEra1o@outlook.es (V.S.S)

* Correspondence: cibis_herj@yahoo.com.mx; Tel.: +52-777-361-215-5

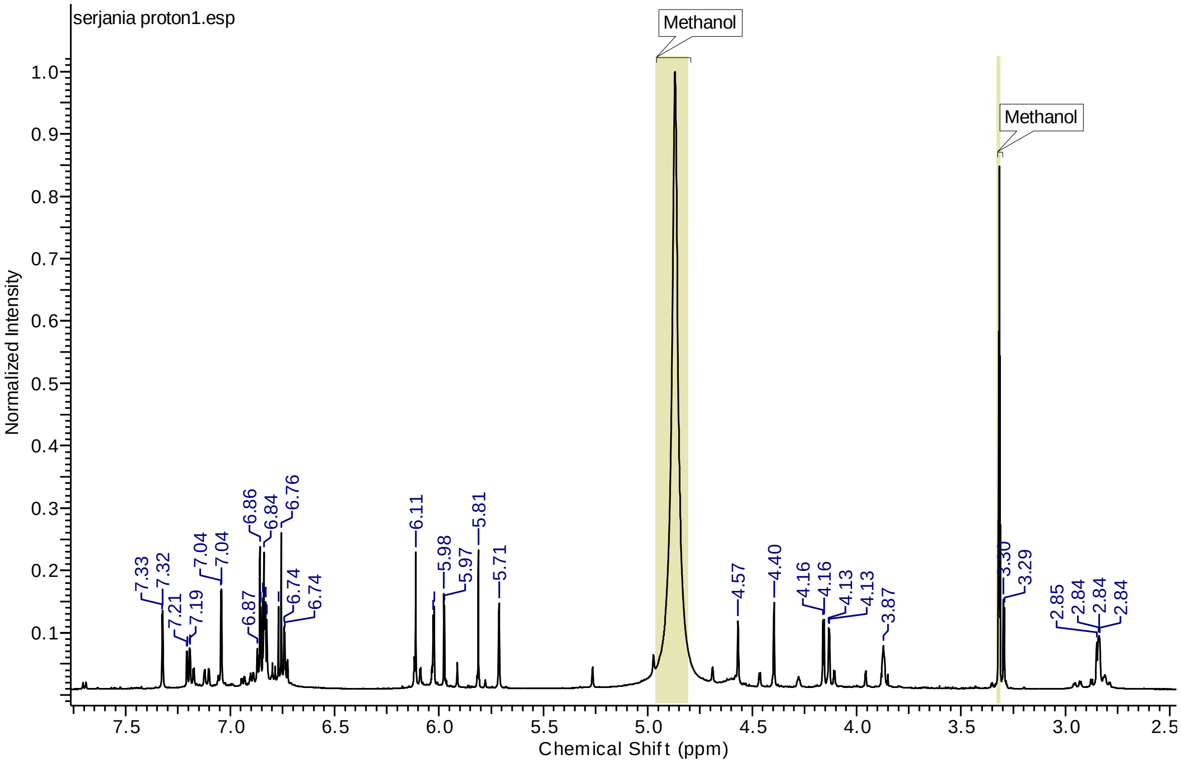

Fig. 1S ^{1}H NMR (CD_3OD, 400 MHz) Compound ETP.

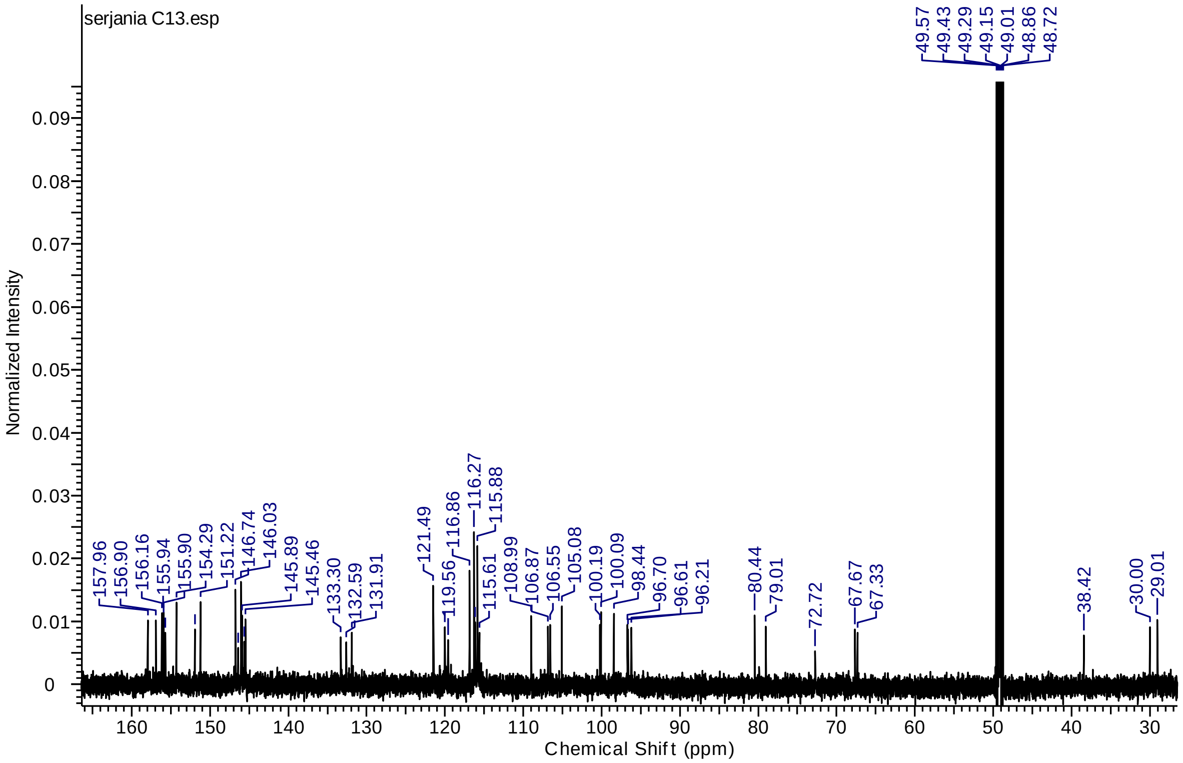

Fig. 2S ^{13}C NMR (CD_3OD, 100 MHz) Compound ETP.

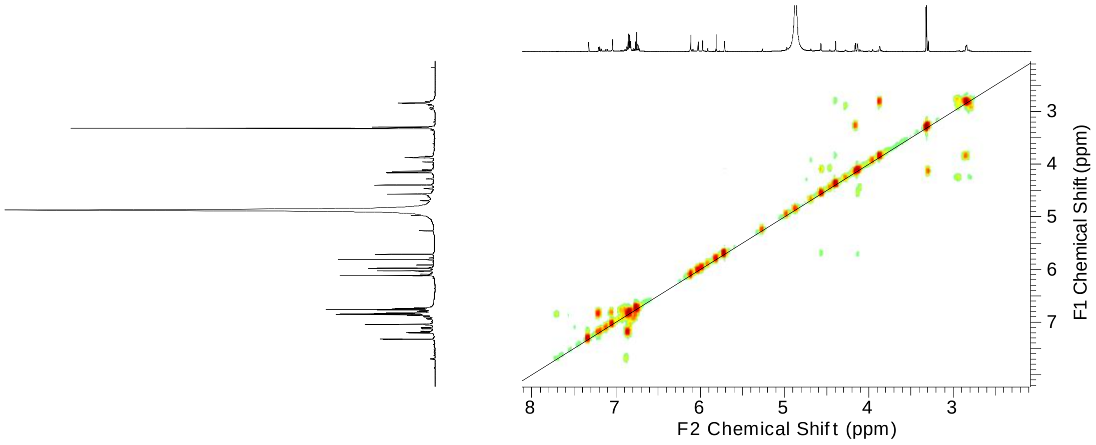

Fig 3S. ^{1}H – ^{1}H COSY (CD_3OD, 400 MHz) Compound ETP.

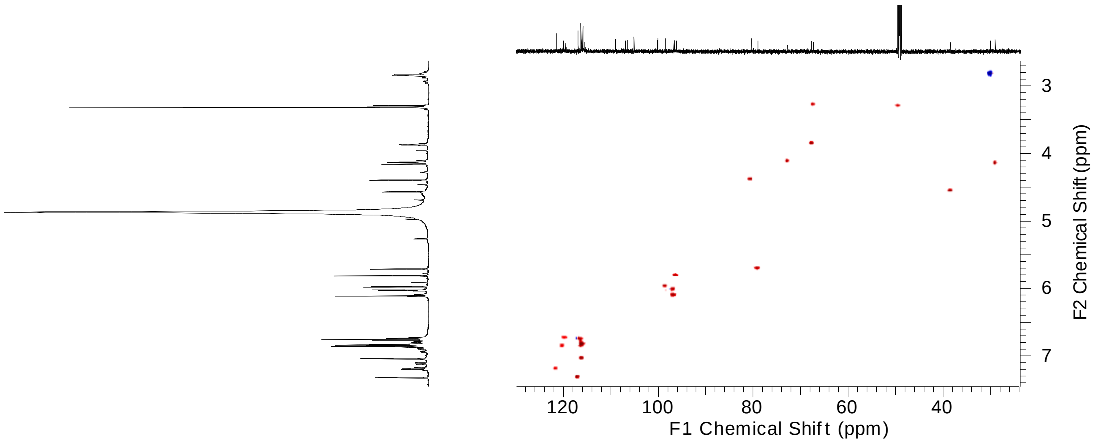

Fig4S. gHSQC (CD_3OD, 400 MHz) Compound ETP

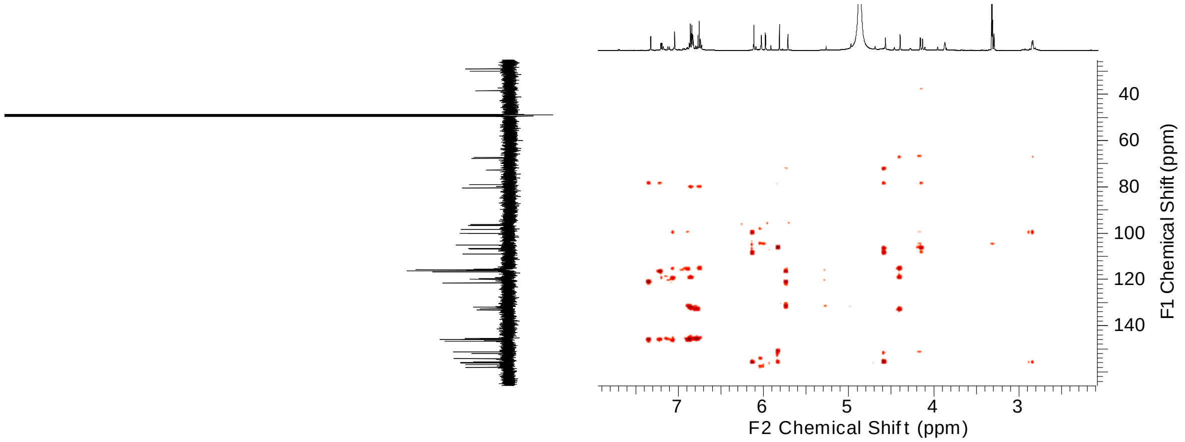

Fig. 5S. HMBC (CD_3OD, 400 MHz) Compound ETP

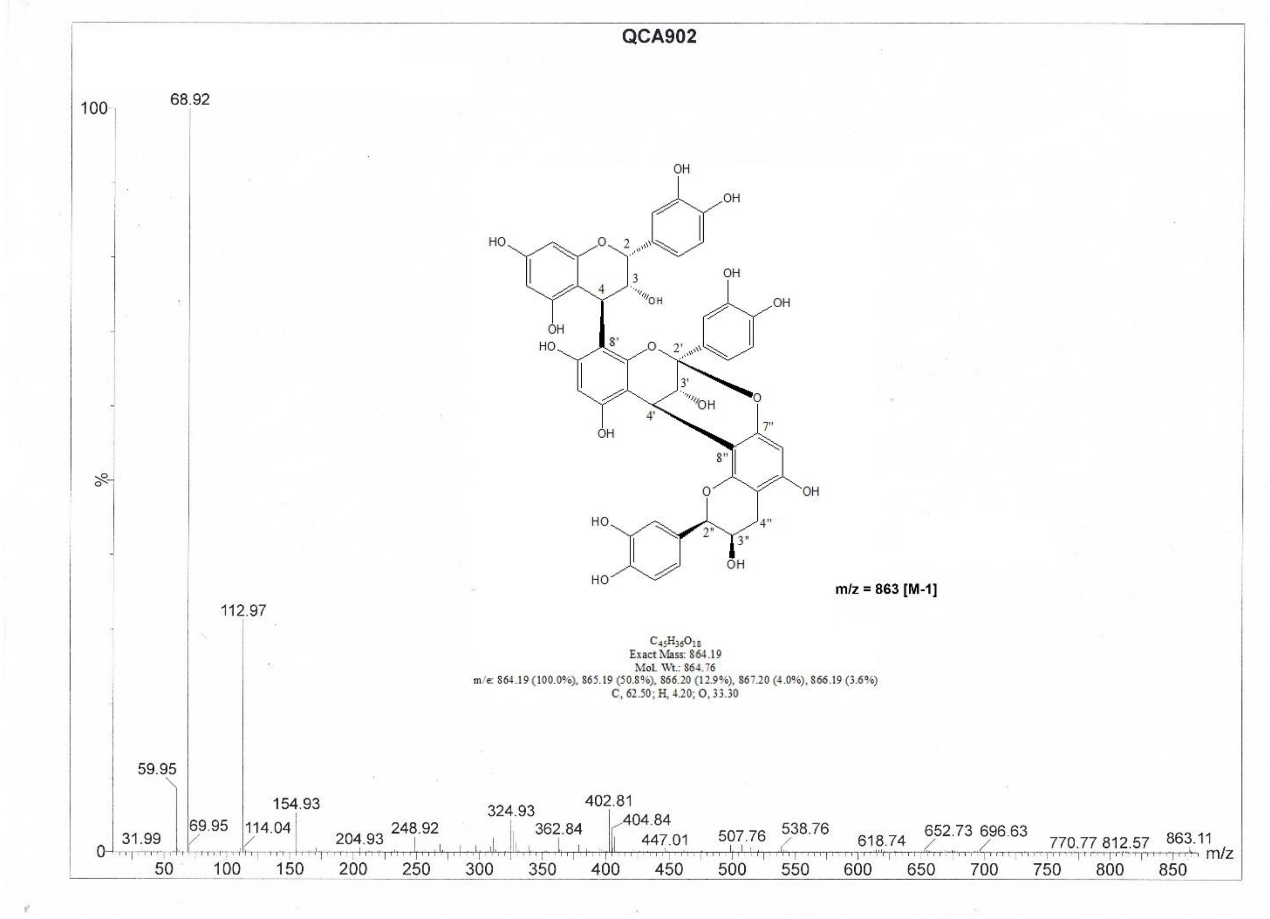

Fig 6S. Mass spectra Compound ETP

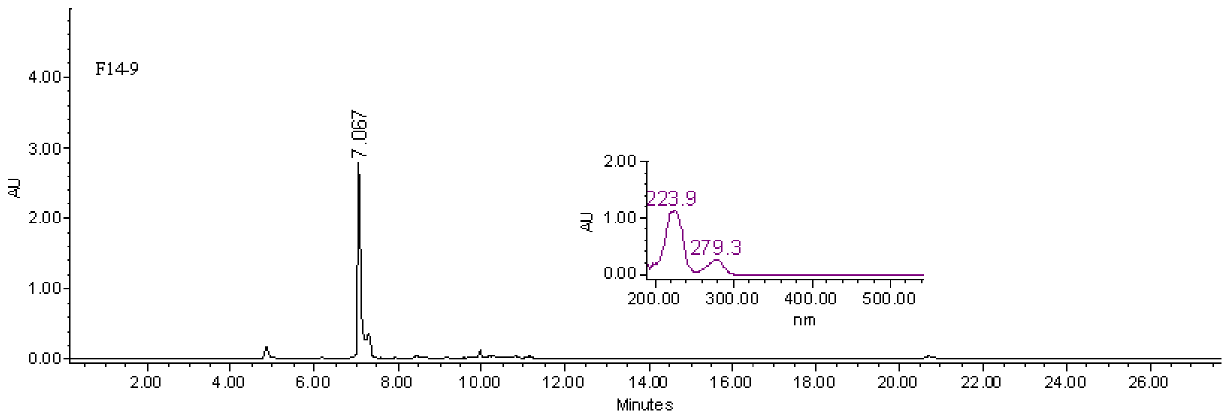

Fig. 7S. HPLC Analysis of Fraction F14-9
